# To alert coinfection of COVID-19 and dengue virus in developing countries in the dengue-endemic area

**DOI:** 10.1017/ice.2020.187

**Published:** 2020-05-04

**Authors:** Di Wu, Jianyun Lu, Qun Liu, Xiaowei Ma, Weiyun He

**Affiliations:** 1 Guangzhou Center for Disease Control and Prevention, Guangzhou, China

*To the Editor—*The SARS-CoV-2 outbreak has raised serious concerns worldwide. The World Health Organization (WHO) has raised the risk of spread to very high level, and as of March 30, 2020, a total of 634,835 cases had been reported, including 29,891 deaths.^1^ Gabriel Yan et al^2^ reported 2 cases of COVID-19 patients coinfected with dengue fever in Singapore. The cases shared similar diagnoses and disease courses. They both first tested negative for dengue using a rapid test, then they were discharged and returned to the hospital for persistent fever and were then diagnosed with dengue fever and SARS-CoV-2 coinfection. Joob et al^3^ also reported a patient coinfected with SARS-CoV-2 and dengue virus in Thailand. This patient first presented with a with petechiae skin rash and was diagnosed with dengue fever. However, the patient further presented with respiratory symptoms and was rediagnosed with COVID-19 infection. These 3 cases raise concern that patients with fever can be infected with both SARS-CoV-2 and dengue at the same time in dengue-endemic areas such as Singapore, Thailand, and Malaysia in Southeast Asia and Brazil in South America. According to a recent study of 1,099 patients conducted by Guan et al,^4^ 87.9% of COVID-19 patients present with fever, 67.7% present with cough, and 13.7% present with headache. Some patients present only with fever when infected with SARS-CoV-2. In another study of 1,792 patients, 100% of dengue fever patients presented with fever and 25.7% presented with headache.^5^ Thus, COVID-19 patients can present the same clinical signs as dengue patients. Furthermore, the Singapore cases were misdiagnosed and later confirmed with COVID-19,^2^ which shows that the misdiagnosis of the patients with atypical symptoms (as listed above) is possible. Therefore, measures should be taken to distinguish patients with fever and headache from dengue fever and COVID-19, and these atypical symptoms should trigger alerts, especially in developing countries with a high incidence of dengue fever, as in Southeast Asia and South American. We strongly recommend that rapid, sensitive, and accessible tests include a polymerase chain reaction (PCR) test of nasopharynx swabs and anal swabs. Furthermore, dengue NS1, IgM, and IgG tests should be used to distinguish those with atypical symptoms in the developing countries facing the coming dengue endemic.
